# A mixed-method evaluation of a volunteer navigation intervention for older persons living with chronic illness (Nav-CARE): findings from a knowledge translation study

**DOI:** 10.1186/s12904-020-00666-2

**Published:** 2020-10-15

**Authors:** Barbara Pesut, Wendy Duggleby, Grace Warner, Paxton Bruce, Sunita Ghosh, Jayna Holroyd-Leduc, Cheryl Nekolaichuk, Jasneet Parmar

**Affiliations:** 1 University of British Columbia Okanagan, 1147 Research Road, Arts 3rd Floor, Kelowna, BC V1V 1V7 Canada; 2 University of Alberta, 3-141 ECHA 11405 87th ave, Edmonton, Alberta Canada; 3 School of Occupational Therapy, Dalhousie University, P.O. Box 15000, Halifax, Nova Scotia B3H 4R2 Canada; 4 University of British Columbia Okanagan, 1147 Research Road. Arts 3rd Floor, Kelowna, BC V1V 1V7 Canada; 5 University of Alberta/Alberta Health Services, 11560 University Ave, Edmonton, AB Canada; 6 University of Calgary, 1403 29th Street NW, Calgary, AB Canada; 7 Department of Oncology, University of Alberta, c/o Palliative Institute, Health Services Centre, DC-404, 1090 Youville Drive West, Edmonton, AB Canada; 8 Specialized Geriatrics Program, Department of Family Medicine University of AB, Medical Lead, Home Living and Transitions, AHS EZ Continuing Care, c/o Grey Nuns Community Hospital, 416 St. Marguerite Health Services Centre, 1090 Youville Drive West, Edmonton, AB T6L 0A3 Canada

**Keywords:** Volunteers, Palliative, Older persons, Navigation, Hospice, Quality of life, Family caregivers

## Abstract

**Background:**

Volunteer navigation is an innovative way to help older persons get connected to resources in their community that they may not know about or have difficulty accessing. Nav-CARE is an intervention in which volunteers, who are trained in navigation, provide services for older persons living at home with chronic illness to improve their quality of life. The goal of this study was to evaluate the impact of Nav-CARE on volunteers, older persons, and family participating across eight Canadian sites.

**Methods:**

Nav-CARE was implemented using a knowledge translation approach in eight sites using a 12- or 18-month intervention period. A mixed method evaluation was used to understand the outcomes upon older person engagement; volunteer self-efficacy; and older person, family, and volunteer quality of life and satisfaction with the intervention.

**Results:**

Older persons and family were highly satisfied with the intervention, citing benefits of social connection and support, help with negotiating the social aspects of healthcare, access to cost-effective resources, and family respite. They were less satisfied with the practical help available for transportation and errands. Older persons self-reported knowledge of the services available to them and confidence in making decisions about their healthcare showed statistically significant improvements (*P* < .05) over 12–18 months. Volunteers reported satisfaction with their role, particularly as it related to building relationships over time, and good self-efficacy. Volunteer attrition was a result of not recruiting older persons in a timely manner. There was no statistically significant improvement in quality of life for older persons, family or volunteers from baseline to study completion.

**Conclusions:**

Findings from this study support a developing body of evidence showing the contributions volunteers make to enhanced older person and family well-being in the context of chronic illness. Statistically significant improvements were documented in aspects of client engagement. However, there were no statistically significant improvements in quality of life scores even though qualitative data illustrated very specific positive outcomes of the intervention. Similar findings in other volunteer-led intervention studies raise the question of whether there is a need for targeted volunteer-sensitive outcome measures.

**Supplementary information:**

**Supplementary information** accompanies this paper at 10.1186/s12904-020-00666-2.

## Background

Finding innovative ways to care for a population aging with complex, chronic illness is high on the healthcare agendas of developed countries [1–3]. An area of particular concern is the need to close gaps in support for those transitioning from chronic illness management to palliative care [4, 5], a transition period referred to in this study as *advanced chronic illness*. Evidence describing the struggles of older persons living at home with advanced chronic illness is compelling [6, 7], illustrating the urgent need for enhanced support. These older persons often live with heavy symptom burden [8–10] and are at risk for social isolation [11]. Their need for support, information, advocacy, and assistance with decision-making is high [12]. They may not know of the health or social services available in their community [13]. Indeed, this time on the palliative trajectory may be more problematic than the actively dying phase due to the lack of suitable supports [14].

Persons living with advanced chronic illness are an early palliative population; they are not imminently dying, but death within a year would not come as a surprise [15]. A palliative approach, in which the supportive principles of palliative care are used early in the palliative trajectory [16, 17], has been used to describe ideal care for this population [4, 18–20]. However, little evidence exists on how to realize this ideal of early supportive care [21]. One cost-effective way to provide early palliative support is through volunteers. Hospice volunteers have traditionally played a unique intermediary role with patients and can provide support and practical help [22]. Hospice and palliative care societies have long recognized the importance of early support [23], and hospice volunteers are ideally positioned to contribute to this support [24], but instead they are often introduced late in the palliative trajectory [25]. When this happens, an important opportunity to enhance high quality palliative care is lost.

Another innovative way to provide support for older persons living with chronic illness is patient navigation [26, 27]. A robust body of evidence indicates that navigation is effective in improving cancer treatment by facilitating access to services [28, 29]. Volunteer and peer navigation have been important aspects of this agenda [30]. Navigation has also been used to reduce hospital readmissions for older persons [31]. Navigation seeks to connect persons to resources that they might not otherwise be aware of and helps remove barriers to accessing those resources. Our early ethnographic work in rural palliative care revealed that many resources in rural communities were underutilized because persons who needed them did not know they existed [32, 33]. Further, we learned that whereas healthcare professionals assisted with healthcare navigation, there was a gap in assisting individuals to get access to practical helps that had the potential to significantly improve quality of life [34]. Volunteer navigators have the potential to be a cost-effective way to address this gap.

Other recent innovations have used trained volunteers to improve the care of older persons living in community. A Canadian program called Tapestry, designed to improve healthy aging, used volunteers to assist older persons with goal setting and to collect person-centred data for the interdisciplinary team. Tapestry focused on older persons who were not yet in their last year of life. A pragmatic randomized controlled trial of the Tapestry intervention failed to show any significant differences on study outcomes related to older person goal attainment; however, qualitative data collected from older persons participating in the intervention indicated positive responses to the volunteer visits [35–38].

The End-of-Life Social Action Study (ELSA) in the United Kingdom used trained volunteers to provide face to face support (befriending, practical support, and signposting) to persons at home in the last year of life. Volunteers conducted weekly visits, lasting 1–3 h, over a four-week period. A pragmatic randomised wait-list trial failed to find significant differences in quality of life, loneliness or social support between the treatment and control groups. Qualitative case study analysis of the intervention suggested that the impact of the intervention could be enhanced by ensuring that volunteers take a relational and goal-based focus in their role [39–41].

Based upon the foundations of navigation and a palliative approach to care described above, and upon a series of studies we conducted exploring issues and potential solutions in rural palliative care [42–46], we designed a volunteer-led navigation intervention, called Nav-CARE (Navigation-Connecting, Accessing, Resourcing, and Engaging).

*Navigation is defined as working in collaboration with patients, families, and communities to: a) negotiate the ‘best fit’ for the needs of persons, their families, and communities and resources; b) improve access to needed services and resources at the end of life (including death) and bereavement; and c) promote quality of life, foster independence, and facilitate community connections utilizing a culturally safe, palliative approach* [47 p. 1]*.*

Nav-CARE helps older persons living at home with advanced chronic illness to gain social support and find resources to maintain their independence and meet their needs. Volunteer navigators are trained to become friends and advocates and to create connections with community-based supports and resources. It is uniquely adapted to care within community using the appropriate scope of volunteers. It is important to note that Nav-CARE was designed to enhance, not replace, professional health and social care navigation.

The study reported here builds upon the culmination of collaborative work that entailed developing the conceptual and theoretical foundations for older person navigation [47, 48]; creating, testing, and refining curriculum for volunteer navigators [49]; and conducting three incremental pilots to determine the feasibility and acceptability of Nav-CARE [50, 51]. These pilots provided care to older persons living with a variety of advanced chronic illnesses, of which cancer was the most prevalent. Outcomes from a 3-year pilot (2011–14) indicated that the use of a rural nurse navigator was effective in meeting the needs of this population [50]. Results from two pilots conducted in rural Alberta and British Columbia (2015–16) to test the feasibility and acceptability of adding trained volunteer navigators in partnership with the nurse navigator showed similar positive results. Volunteers were successful in facilitating navigation services, felt well-prepared and were satisfied in the role [51]. Older persons and family who were interviewed indicated that the service was important to their care. They cited specific benefits of having a volunteer navigator such as assistance with decision-making, the presence of a social safety net, and improved engagement with life which in turn influenced their perceptions of their illness experience [51]. These pilots indicated that Nav-CARE was a feasible and acceptable way to support older persons and their family living in community with advanced chronic illness.

Based upon these pilots, the purpose of this project was to further develop Nav-CARE by implementing, evaluating, and sustaining it in eight Canadian communities using an integrated knowledge translation approach. The objectives of the knowledge translation study were two-fold: (1) to better understand the factors that influence implementation and sustainability and (2) to evaluate the impact of the Nav-CARE program using a mixed-method approach. This manuscript focuses on objective 2, evaluating the impact of the Nav-CARE program. A related manuscript focuses on objective 1 by describing Nav-CARE implementation and sustainability issues across diverse study sites [52].

## Methods

### Aim/design/setting

Impact was evaluated using a mixed-method design of concurrent triangulation [53] in which qualitative and quantitative data were collected concurrently to overcome the limitations of a single approach. From pilot studies we knew that participants could speak eloquently to the effect of the intervention on their lives, thus providing us with an interpretive account of impact. We further sought to confirm those findings through quantitative measures. Qualitative data was collected through semi-structured interviews. Quantitative data was collected through pre- and post-test measurements.

The setting was eight community-based hospice societies located in British Columbia (*n* = 6), Alberta (*n* = 1), and Nova Scotia (n = 1). Table 1 provides information about the communities in which these hospice societies were located.

**Table 1:** Communities in Study

	Population	Distance to Urban^a^ (km)	% population > 55	% population unattached^b^
1	9000	60	38.1	27
2	48,000	51	40	31
3	10,000	350	33	37
4	150,000	NA	36	28
5	5000	60	62	35
6	12,000	90	25	21
7	19,000	400	35	29
8	10,000	400	29	33

^a^Population of greater than 100,000

^b^Persons not living in an economic family (Stats Canada)

### Participant recruitment

Hospice societies were recruited through pre-existing relationships. Using an integrated knowledge translation approach, leaders from these societies assisted with framing the study and were partners on the grant application. To be eligible for participating in the study, hospice societies were required to create a Nav-CARE advisory committee and to designate a Nav-CARE volunteer coordinator who would be responsible for site-specific Nav-CARE duties. Volunteers were recruited through the Nav-CARE coordinator at each study site. To be eligible for the study volunteers were required to have hospice or equivalent volunteer training, participate in an interview to determine suitability for the role, and undergo a criminal record check. Older persons and family living with advanced chronic illness were recruited by the volunteer coordinator from the community through healthcare providers, public advertising, and word of mouth. Older persons were eligible for the study if they were living with a chronic illness that could reasonably lead to death within the next year and if they had the cognitive capacity to fill out the study measures. Stipends were provided for the volunteer coordinator and volunteers out of study funds to help offset some of the costs related to participating in the research.

### Nav-CARE intervention

An implementation toolkit that provided detailed start-up instructions was provided to each community. After the advisory committee was established, the volunteer coordinator appointed, and the volunteers recruited, members of the research team provided 14 h of in-person training in navigation. Volunteers and volunteer coordinators attended the training. One of the research team members was an advanced practice nurse who performed a nurse navigator role and a volunteer mentor role in the early pilots of the Nav-CARE intervention [50, 51]. After the volunteer education, older persons were recruited and matched with volunteers by the volunteer coordinator. Volunteers were instructed to visit with their clients biweekly, although the frequency and nature of the visits were to be negotiated between the volunteer and the older person. Visits were conducted in-person or by phone depending upon the preferences of the older person. The intervention period was either 12 or 18 months.^1^ Coaching sessions were provided to volunteers via teleconference every four weeks by the advanced practice nurse who conducted the training. Volunteer coordinators participated in teleconferences every six weeks to share implementation experiences.

### Data collection

Data was collected between December 2016 and February 2019. As this was a rolling intervention across study sites, data collection was mapped from the date of volunteer education. Three time points were used because we anticipated that some older adult participants would die before the study concluded and thus, it was important to have midpoint data. Further, midpoint data on volunteer self-efficacy allowed us to determine areas for volunteer development. These areas for development were integrated into the mentorship sessions. The following data was collected: (1) Older person and family satisfaction was evaluated through semi-structured interviews at the midpoint of the intervention (i.e., 6–9 months). Interviews explored why older persons and family participated in Nav-CARE, benefits they incurred, and suggestions for change. (2) Older person’s engagement was evaluated through a questionnaire completed at the beginning, midpoint, and end of the intervention. Older persons were asked to respond to 12 items exploring knowledge of resources, confidence in decision-making and communicating those decisions, and social support using a Likert scale from 1 (all of the time) to 5 (none of the time). Items were derived both from the goals of the intervention (e.g., access to information) and from the benefits of having a volunteer navigator described by older persons participating in the pilot work (e.g, assistance with decision-making). The questionnaire was developed and validated during the pilot studies (see Supplementary File 1). Items were derived from the perceived benefits of the intervention as described by older person participants and family. Face validity was established by having several experts in the Nav-CARE intervention review the items. The questionnaire was then trialled on a sample of older persons to ensure that items were easily understood. Analysis of the questionnaire results revealed little missing data suggesting that older persons were understanding the items. Descriptive statistics showed good variability in the item scores revealing a range of response options. No further psychometrics were conducted. (3) Volunteer self-efficacy in navigation and role satisfaction: A questionnaire to measure self-efficacy in navigation was developed for this study based upon the competencies developed by the principal investigators in a previous study [48]. These competencies informed the development of the curriculum for volunteer preparation. Volunteers were asked to report their self- efficacy on 32 competencies, using a scale of 0 (not at all confident) to 5 (highly confident) immediately after the training workshop, at the midpoint of the intervention, and at the conclusion of the intervention. Possible scores ranged from 0 to 160 with higher scores indicating higher self-efficacy. The questionnaire was developed and validated in the pilot study (see Supplementary File 2). (4) Volunteer satisfaction was measured at the end-point of the intervention using a 43-item questionnaire that inquired about satisfaction on a 5-point Likert scale from 0 (strongly disagree) to 5 (strongly agree) on orientation (4 items), training (8 items), performance feedback (9 items), communication (7 items), social contacts (4 items), and value and respect (11 items). This questionnaire was adapted for Nav-CARE with permission from a previously developed and validated hospice volunteer satisfaction questionnaire [54]. Possible scores ranged from 43 to 215 with higher scores indicating higher satisfaction (see Supplementary file 3). (5) Older person, family, and volunteer quality of life (QOL) was measured at the beginning, midpoint, and end of the intervention using the SF12v2, a well-validated measure with established norms for the general population [55, 56]. Questionnaires were distributed by volunteer coordinators, completed confidentially by the participants and returned to the research office via mail for analysis.

Semi-structured interviews with older persons, family, and volunteers were completed over the telephone by the research coordinator and were audio recorded and transcribed verbatim by a research assistant or transcriptionist. The interview guide, which was adapted for the different participants, focused broadly on reasons for participating in Nav-CARE, experiences with and perceived benefits of the service, challenges encountered, and suggestions for improvement. The interview guide was piloted prior to use.

### Data analysis

Qualitative data were managed through NVivo qualitative software and analyzed using qualitative descriptive techniques [57]. A codebook was developed based upon the interview questions that broadly explored successes, challenges, and recommendations for change of the Nav-CARE intervention. Preliminary coding was completed by two investigators to ensure coding integrity. A thematic account was developed for each group (e.g., older persons, family, volunteers). Data analysis and collection occurred concurrently; however, no substantive changes to the interview guide were made based upon findings.

Quantitative data was entered into SPSS and cleaned. Descriptive statistics were run for all measurement instruments. Mean, range (min and max) and standard deviation were reported for continuous variables. Frequency and proportions were reported for the categorical variables. The satisfaction and self-perceived competency questionnaires were analyzed using total scores. The engagement questionnaire was analyzed by item. The SF12v2 was analyzed and reported as a physical component (PCS) and a mental component summary (MCS).

Generalized estimating equation (GEE) method was used to analyze the longitudinal data. GEE method provides parameter estimates and robust standard error accounting for the within and between subject variability. The standard statistical methods does not account for the within subject variability arising from repeated measures on the same individual overtime, hence GEE method was used. One of the major problem of longitudinal data is missing data overtime. Multiple imputation (MI) method was used to impute for missing data. The MI method uses Markov Chain Monte Carlo (MCMC) algorithm known as chained equation imputation. A maximum of 5 imputed datasets were created and 200 iterations separating each imputed dataset were used. The imputed values were compared with the non-imputed values and the results were very similar, indicating that the missing data was not associated with the outcome variable and missing at random (MAR). The results from the pooled analysis (average of results from 5 imputed dataset) were presented. The analysis was conducted for the client engagement data and quality of life data. SPSS version 25 (IBM Corp. Released 2017. IBM SPSS Statistics for Windows, Version 25.0. Armonk, NY: IBM Corp) was used to analyze the data and a *p*-value < 0.05 was used for statistical significance. A two-sided test was used for the analysis.

## Results

**Fig. 1:**
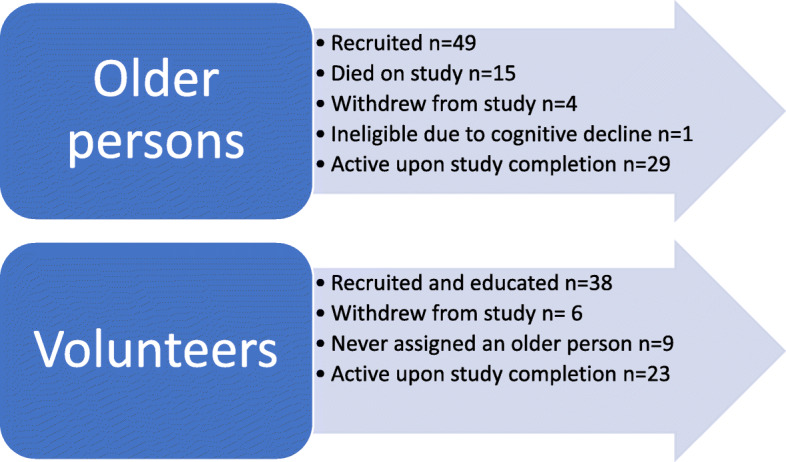
Study Participant Recruitment. This figure provides a breakdown of the numbers of older persons and volunteers recruited, numbers who were active at study completion, and reasons for study attrition

Participants included older persons (*n* = 49) living in community with advanced chronic illness, family members (*n* = 18), and volunteers (*n* = 38) who delivered the intervention. (See Table 2 for demographic information). Fifteen older persons died during the study intervention, 4 older persons withdrew, and 1 older person was unable to continue due to cognitive decline. Further, there were delays in recruiting older persons and additional persons were recruited to replace attrition from death and withdrawal. This meant that not all participants received the full 12–18-month intervention. Six volunteers withdrew from being a Nav-CARE volunteer, and 9 were never assigned to an older person, and hence, were inactive. The most significant reason for volunteer attrition was failure to recruit older persons in a timely manner [52]. Twenty-three volunteers completed the study (See Fig. 1).

**Table 2:** Demographic Information of Study Participants

Volunteer Demographic Information *N* = 38		
Age	Range: 30–78	
	Mean 61.84 (SD 10.7)	
Employed	No 30 (78.9%)	
	Yes 8 (21.1%)	
Years of Volunteer Experience	0–5: 5 (13.2%)	
	6–10: 9 (23.7%)	
	> 10: 24 (63.2%)	
Older Person/Family Demographic Information		
	Older Persons (*n* = 49)	Family (*n* = 18)
Age	Range: 55–95	Range: 46–86
	Mean: 71.84 (SD 11.35)	Mean: 65 (SD 12.93)
Sex	Male 17 (34.7%)	Male: 5 (27.8%)
	Female: 32 (65.3%)	Female: 13 (72.2%)
Relationship to Older Person		Partner: 14 (77.8%) Child: 4 (22.2%)
Receiving other services	Yes 22 (44.0%) No: 27 (55.1%)	
Living Arrangements	Home alone: 23 (46.9%) Home with family 22 (44.9%) Other: 4 (8.1%)	
Number of chronic health conditions	1: 25 (51%) 2: 14 (28.6%) 3 or more: 10 (20.4%)

In total, the volunteers over all sites during the study time period visited older persons 720 times. On average the volunteers visited older persons for 77.54 min (SD 41.27) (see Table 3) with 77% of visits taking place in homes [557/720]. In reporting this evaluation, we will first present older person and family satisfaction as derived from the qualitative data and the quantitative results of the older person engagement measure. We will then present a synthesis of qualitative and quantitative findings of volunteer self-efficacy and role satisfaction. Finally, we will present findings of older person, family, and volunteer quality of life as measured by the SF12v2. Codes for the quotes derived from participants are as follows: P = older person, F = family, V = volunteer.

**Table 3:** Visit Statistics (*n* = 720 visits)

Average Length of Visit in minutes	Mean 77.54 (SD 41.27)
Type of Visit	Scheduled: 597 (83%)
	Unscheduled: 123 (17%)
Place of Visits	Home: 557 (77%)
	Hospital: 25 (3.3%)
	Long term care: 66 (9.2%)
	Other (e.g., phone, coffee shop): 73 (10%)
Days Between Visits	Range: 1–117^a^
	Mean: 18.4 (16.3)
Time spent on navigation activities between visits in minutes	Mean 32.32 (SD 42.73)

^a^The reflects a client who take a break from receiving services

### Older person and family satisfaction

Twenty-one older persons and 11 family members participated in satisfaction interviews. During this interview, older persons were asked to rate their satisfaction with the Nav-CARE program and their perception of its importance on a scale of 1 (not important) to 10 (very important). All older persons who responded (20/20) rated their satisfaction with the program as 8 to 10 out of 10. The majority of older persons (17/19) rated the importance the program as 8 to 10 out of 10. Six of 10 family members who completed the survey rated the importance and their satisfaction with the program as 10 out of 10. Qualitative data suggested that Nav-CARE was perceived to be important because it helped with social connection and support, with navigating the complexities of healthcare, and with cost-effective access to resources. Family members cited additional benefits of emotional and physical respite. However, participants were less satisfied with the volunteer’s inability to provide transportation or to perform errands.

#### Social connection and support

Older persons emphasized how the program provided social support, especially for individuals living alone or in isolated situations. “*My two boys are in the city and especially in the winter, they can’t come down because they have their own jobs and families to look after. So, it was nice to have someone in the community come to talk to me”* (P47). They further spoke of the value of having a supportive friend who checked in on them regularly. “*It was just knowing that I could pick up the phone and call her, I guess, that was the most important”* (P30). Another older person who felt isolated remarked on the sense of self-worth she felt when the navigator visited. *“Sometimes I sit here and feel sorry for myself. But after she leaves, I feel kind of important to have somebody like her come and visit me”* (P51).

In addition to social support, older persons spoke of how the navigator taught them special skills, listened to their concerns, and engaged them in enjoyable activities. One described how her navigator taught her to cope well with stress, to listen to music and to just let go, and how this guidance was benefiting her health condition. *“I’m so glad I signed up. I look forward to her visits, and that helps me. After she leaves I just feel so much better”* (P36). For another individual, it was having a confidante that made the relationship important. “*I’ve learned to live with what I have and I don’t talk about it all the time. But to be able to talk to someone who understands you, really understands what you’re going through, has been a relief to me”* (P37). The importance of having someone listen to their concerns was echoed by others. “*I have a lot of friends and at first I thought the service would be a waste of time. But my navigator has talked me through things I could not tell friends and family and has helped me figure out what they need to know”* (P29). Another person, self-described as *“not a loner”* commented on how much he enjoyed going to a ball game with his navigator, an outing that went beyond the usual services provided by the program (P41). The social aspect of the program was so valued that older persons mentioned when their navigator took holidays, a replacement volunteer should be sent to call or visit them. This social aspect led one individual to reflect on the value of the program. “*It’s not focused on dying. It’s focused on living - living the best you can with whatever you’ve got each day of the year. And so, that’s been a real bonus for me from this research. It’s led to other connections”* (P37).

Like older persons, family remarked on the value of social support for their own needs as caregivers. “*When you’re feeling really low, like, oh, my gosh, how much more can I take? Then, she would build you up. She would go, you know, ‘You’re doing something amazing and, you know, you’re making a difference in your mom’s life.’”* After the older person passed away, the family stayed in touch with the navigator saying, *“the role was still valuable” (F33).*

#### Negotiating social aspects of healthcare

Older persons spoke of how volunteer navigators supported them to negotiate the complexity of modern healthcare. They did so through a relational approach that enabled them to identify resources and make sense of healthcare information. Volunteers were perceived as reliable and understanding, two qualities older persons believed were missing in modern healthcare. For example, one person shared how important his navigator’s punctuality was to him. “*I need something that’s reliable. She’s usually a minute early or once in a while she’ll be a minute late. When you’re in this condition, you really need stability and security. It’s really been profound. I’ve told her, but I haven’t been weeping when I told her. I’m getting all weepy as I think about this”* (P12). Another person suggested that the navigator may have a better understanding of what they are going through than a healthcare professional. “*You need someone who understands and is supportive. And, you know, you can’t rely on doctors. Most of them have never been sick in their life. They don’t understand the amount of pain you go through”* (P37). An older person who was intensely frustrated by medical professionals said his navigator helped him deal appropriately with the dilemma by letting him discuss it. “*When I described my frustration with my doctor she just supported me by saying, ‘that would be frustrating.’ She just provided support. So, I looked at my options and I worked it through on my own. That was really key, that changed the power dynamics. Rather than feeling like a victim of a doctor, I felt like, okay, this is where it changes”* (P12).

Older persons suggested that volunteers played an important role in helping them identify relevant resources in the community and make sense of healthcare appointments and decisions. Navigators identified credible medical information for their clients helping them better understand their health condition. One older person, self-described as “*medically illiterate*”, reflected on how his navigator helped him to understand his medical condition and the purpose of various tests and treatments (P12). The navigators’ visits were a chance for older persons to debrief after medical consults, emergencies, or tests. One individual talked about how her navigator helped her find a neurologist closer to home (P29). Others explained how their navigator could identify when they needed to consult with a doctor about their condition and then coach them in what to say and questions to ask (P37).

Likewise, family described similar benefits from having a navigators’ support through the healthcare journey. “*Having her alongside is good. She asked if she could come to the radiologist. And she was there and, you know, she’s kind of like a third ear, right? Like a third ear.”* (F34).

#### Access to cost-effective resources

Older persons valued the practical ways in which the navigator helped them to access low cost services in their community. For example, one person whose navigator helped her complete a will with pro bono legal services, also listed benefits such as getting a handyman to do repairs and finalizing a divorce in a cost-effective manner (P39). Another individual detailed how her navigator connected her to an organization that could help pay for her medications and introduced her to a free day program at hospice where she could meet others and engage in pleasurable activities (P37). One person described how her navigator found a grocery store in their community that delivered to seniors for free, introduced her to a website with seniors’ resources for home maintenance and chores at lower costs, and got her enrolled in a subsidized wheelchair transportation program (P29). An older person whose husband was deaf and near-blind appreciated the information her navigator provided about housing, meals, and other resources that the couple needed to remain independent. This individual explained, *“anything that came up, she would say, I’ll look into that for you, and she did”* (P32).

#### Family respite

In addition to the benefits described above, family members specifically mentioned the physical and emotional respite they received through the volunteer navigator visits. For example, this family member described the value of physical respite. “*I really feel that if I asked her to drop over for a few hours while I’m at a doctor’s appointment, or I’m somewhere for whatever reason, that she would accept it”* (F42). This was particularly helpful if the family member reported that the older person enjoyed the visit. *“It gives him something to look forward. And then, if I need to go into the drug store or to the store or something, I know I don’t have to worry about him”* (F41). For some family members, the navigator visit brought a welcome change from isolation. *“My wife seemed to be a little more outgoing in those visits than just locked up in the house with me. It was a difficult time and the volunteer certainly assisted me and my two daughters by sharing the load”* (F26). Respite for some family took the form of letting the navigator take over medical conversations so they could maintain some measure of their former life. *“I just wanted to be the daughter. I didn’t want to be the nurse. And the decisions about medications. If I had to talk any more about those, oh my God”* (F33).

#### Nav-CARE gaps: transportation and errands

There were also significant gaps identified by older persons and family members in what Nav-CARE was able to provide. Many wished the navigator role could include providing transportation to appointments, shopping, or social outings. Further, they wished for spontaneous help with errands. “*I would want the workers to be more relaxed about offering, ‘Oh, I’ll be out in the community. Can I pick anything up for you?’ This is a small community and it would be easy for them to do that”* (P12). This type of help was particularly important for those individuals who were experiencing declining mobility or were housebound.

### Older person engagement

At the conclusion of the intervention, older persons indicated moderately high (defined as means of less than 2 on a 5-point scale) in response to statements asking if they have people to turn to when they need help; they have confidence in contacting someone when they have a health problem; and they feel confident in communicating their needs and wishes to their doctor (See Table 4). However, there were few statistically significant changes on individual items between Time 1, Time 2, and Time 3. (See Table 5 GEE analysis) Participants’ self-reported knowledge of the services available to help them showed statistically significant (*P* < .05) improvements between Time 1, Time 2, and Time 3. Further, participants felt more confident making decisions about their health and healthcare from Time 1 to Time 2. However, participants also reported feeling less confident in making decisions about their life changes and in communicating their needs and wishes to their doctor at Time 3.

**Table 4:** Older Persons Engagement: Unadjusted Mean Scores

ITEM	T1 *n* = 48 Mean (SD)	T2 *n* = 33 Mean (SD)	T3 *n* = 21 Mean (SD)
1. I feel I know the services available in my community to help me	2.83 (.95)	2.30 (.95)*	2.05 (.74)*
2. I feel like I have people I can turn to when I need help	2.02 (1.00)	1.79 (.86)	1.67 (.66)
3. I feel lonely (reverse scored)	3.81 (1.05)	4.09 (1.07)	3.6 (1.19)
4. I feel I can be involved in the things that are important to me	2.60 (1.05)	2.79 (1.17)	3.1 (1.2)
5. I feel I have someone I can talk to about the things that are troubling me	2.00 (1.09)	2.00 (.87)	2.14 (1.01)
6. I feel confident in making decisions about my life changes	1.9 (.83)	1.88 (1.02)	2.10 (1.04)*
7. I know where to get information about my illness	2.04 (.97)	1.79 (.89)	2.10 (1.07)
8. I feel confident in taking care of my illness	2.13 (.85)	1.84 (.81)	2.2 (.89)
9. I am confident contacting someone when I have a health problem	1.64 (.61)	1.58 (.66)	1.9 (.97)
10. I understand the information given to me by my doctor and other healthcare providers	2.13 (1.04)	1.76 (.83)	2.15 (.81)
11. I feel confident making decisions about my health and healthcare	1.89 (.90)	1.70 (.73)*	2.2 (.77)
12. I feel confident communicating my needs and wishes to my doctor and other healthcare providers	1.85 (.99)	1.67 (.82)	1.75 (1.07)*

* statistically significant changes at *p* < .05. Lower scores indicate improvements. Higher scores indicate diminished engagement

Likert Scale: 1 = “All of the Time” to 5 = “None of the Time”

**Table 5:** Generalized Estimating Equations (GEE) Comparison Table Engagement Questionnaire

Engagement Questions	Time Points	B	Std. Error	Interval		Sig.
				Lower	Upper	
Q1	T1	0^a^	0	0	0	0
	T2	−0.540	0.2192	−0.985	−0.095	0.019*
	T3	−0.727	0.2555	−1.279	−0.174	0.014*
Q2	T1	0	0	0	0	0
	T2	−0.236	0.1831	−0.597	0.125	0.199
	T3	− 0.069	0.2214	− 0.524	0.386	0.756
Q3	T1	0	0	0	0	0
	T2	0.329	0.2476	−0.229	0.887	0.216
	T3	−1.127	0.5120	−2.313	0.060	0.060
Q4	T1	0	0	0	0	0
	T2	0.119	0.2268	−0.330	0.568	0.602
	T3	0.327	0.6184	−1.240	1.893	0.619
Q5	T1	0	0	0	0	0
	T2	−0.012	0.2227	−0.467	0.442	0.957
	T3	0.706	0.5425	−0.654	2.066	0.245
Q6	T1	0	0	0	0	0
	T2	0.029	0.2083	−0.402	0.459	0.891
	T3	0.547	0.2319	0.081	1.013	0.022*
Q7	T1	0	0	0	0	0
	T2	−0.157	0.1700	−0.500	0.187	0.362
	T3	1.020	0.5300	−0.240	2.281	0.097
Q8	T1	0	0	0	0	0
	T2	−0.310	0.1977	−0.726	0.106	0.135
	T3	0.233	0.3976	−0.714	1.180	0.577
Q9	T1	0	0	0	0	0
	T2	−0.046	0.1587	−0.388	0.296	0.776
	T3	0.579	0.4547	−0.572	1.730	0.256
Q10	T1	0	0	0	0	0
	T2	0.049	0.3479	−0.720	0.818	0.891
	T3	0.135	0.3366	−0.595	0.865	0.696
Q11	T1	0	0	0	0	0
	T2	−0.318	0.1250	−0.566	− 0.070	0.013*
	T3	0.433	0.4670	−0.675	1.540	0.385
Q12	T1	0	0	0	0	0
	T2	−0.234	0.2544	−0.802	0.334	0.380
	T3	0.833	0.3871	0.039	1.626	0.040*

a Set to zero as parameter is redundant

*Significant at *p* < .05

### Volunteer self-efficacy in navigation and role satisfaction

Of the 38 volunteers who took part in the study, 2 reported a low self-perceived efficacy score at Time 1, 1 volunteer reported low self-efficacy at Time 2, and 1 volunteer reported low self-efficacy at Time 3. A low self-perceived efficacy was defined as a total score that reflected a mean of 0–2 on the 5-point scale. Individual item analysis indicated that there were no competencies in which volunteers felt poorly prepared. However, several competencies reflected lower scores overall. These were: advocate to meet client/family need with healthcare professionals; assist client/family to overcome service access barriers; advise client/family on negotiating/advocating for care and services; facilitate strategies for self-navigation; create linkages to local leaders, professionals, and resources; provide family with caregiving support and resources; facilitate beginning discussions with client/family about advance care planning and goals of care; assess client/family service usage.

Volunteers indicated overall satisfaction with Nav-CARE on the satisfaction questionnaire. No satisfaction item scored a mean of less than 3 on the 5-point scale. Total scores ranged from 125 to 202, with means of 172.17 (SD 16.58), out of possible score of 215. Four volunteers indicated that they would like further orientation to community resources and 7 volunteers indicated a need for further training. A number of volunteers expressed a desire for more opportunities for social contact with other volunteers.

Twenty-eight volunteers participated in qualitative interviews, providing additional insights into volunteer satisfaction. Volunteers experienced satisfaction in providing their clients with companionship that alleviated social isolation. Listening, helping clients transition toward dying, and building a unique and reciprocal relationship were important aspects of volunteer satisfaction.

#### Listening

An essential part of companioning was being willing to listen deeply: “*Well, they often tell me that they feel so much better after the end of our visit and really I have to be honest that I haven’t done anything except listen*” (V21). An important aspect of listening was being willing to hear those things that family might not want to talk about. “*It was important for her to have someone to express her feelings to who was not part of her family, someone she could be really honest with and know it was not going any further*” (V21). Volunteers also spoke of the satisfaction they derived from learning as they listened to client stories. “*I enjoy being put in a position of having to really think about what it’s like for an older person who has health issues to contemplate what is coming next for them” (V22).*

#### Transitioning toward dying

As they were hospice volunteers first, these Nav-CARE volunteers derived satisfaction from helping clients transition toward dying. “*I think the best day we had was walking to hospice, which is kind of weird, but it was a beautiful day and a normal thing, and we walked together and just chitchatted. I think it was scary for her to go through that step but it was helpful for her and fulfilling for me*” (V35). Another important aspect of this transition was being willing to consider the reality of death. “*Friends and family want you to get better, they don’t want you to think about the fact that you might be dying; whereas I can look at it from a realistic point of view and be open to her concerns”* (V18).

#### Building unique and reciprocal relationships

Navigators recognized that they straddled the emotional investment of family members and the professional work of a paid care provider, requiring a relationship building of a special kind. Many clients did not have family nearby and so navigators felt they filled a critical gap in family support. Others recognized that the volunteer role was special because it was a gift not an obligation. *“Their family comes and does things because they’re obligated. And the institution does what they’re obligated to do. But I wasn’t obligated. She shared her life with me and I was glad to be a part of it”* (V31). Navigators described their clients as friends and suggested that the relationship had become mutually fulfilling. “*We became very attached to each other. She was older than me, but not that much and it was like we knew each other for a long time” (V28).* Although these relationships between volunteers and older persons took time to develop, they were often long-lasting. “*I’m going to be seeing him every week for the rest of - until one of us goes. That’s what’s happened between us. We’ve developed a real relationship” (V18).*

#### Challenging aspects of the role

As satisfying as developing relationships was for these volunteers, there were also challenging issues related to role fulfillment, boundary setting, and complex family dynamics. Some volunteers felt that they did not fulfill the navigational aspects of the intervention. For example, the same volunteer who reported that they made such a difference through the visit to hospice (V35) expressed frustration that more such opportunities did not arise. Volunteers found they had to set boundaries around how much time they would spend with clients and around what tasks they would or should fulfill. “*She was using a walker and she’d have clothes in the wash downstairs. I thought I could be folding those clothes while I’m sitting here. But you know, I just felt that that wasn’t our job*” (V28). Further, they had to learn to allow clients to set boundaries around visit length and schedule. Those who had backgrounds in healthcare had to determine how and when to incorporate that specialized knowledge into their roles as volunteers. Complex family dynamics were also difficult. A navigator observed that family dynamics shifted when she was visiting her client and how she ended up with a mediating role. She had been told that communication between family members was problematic in her absence; but she was able to act as a type of neutral translator that enabled better listening and understanding to take place in the room (V24).

Despite these barriers, overall volunteers expressed a high degree of satisfaction with the Nav-CARE volunteer role. This volunteer summed up what many described as being a source of satisfaction in their role:

*To me, it adds a richness to my life to meet other people, to journey with them. I know it sounds trite but it's the truth for me …and even though one of my clients died, I found the whole process of being the volunteer navigator and being involved with the family as well, it was very powerful for me. You know, it was and I was involved near the end of her life and went to the funeral.* (V21)

Exit interviews for volunteers who resigned or were inactive revealed that the greatest source of dissatisfaction was not having an older person to work with which was a result of recruitment challenges.

## Older person, family, and volunteer quality of life (QOL)

Quality of life was measured at study initiation, midpoint and endpoint (see Table 6). No statistically significant changes were observed for any category of participants over any of the three time points (Table 7 Results of GEE analysis and Supplementary File 4). Data were collated over all time points for each group and compared to population means. For volunteers, 67% (39/58) of scores were at or above the general population norm on the physical component summary, and 100% scored at or above the general population norm on the mental component summary. For older persons, 16% of scores (14/87) were at or above general population norms on the physical component summary and 66% (57/87) scored at or above general population norms on the mental component summary.

**Table 6:** SF12v2 Quality of Life unadjusted mean scores over three time points for participants

	T1			T2			T3		
	n	PCS Mean (SD)	MCS	n	PCS Mean (SD)	MCS Mean (SD)	n	PCS Mean (SD)	MCS Mean (SD)
Older Persons	34	33.82 (10.66)	48.91 (10.82)	33	34.53 (11.49)	50.18 (10.06)	20	30.49 (7.88)	48.42 (12.24)
Family	11	46.88 (8.18)	44.53 (9.08)	11	42.38 (8.97)	45.53 (9.01)	6	44.99 (9.30)	48.65 (7.27)
Volunteers	23	51.91 (8.97)	55.75 (4.17)	23	48.67 (11.72)	56.29 (4.68)	12	49.52 (9.37)	54.40 (6.11)

*PCS* Physical component summary, *MCS* Mental components summary

**Table 7:** Quality of Life General Estimating Equations for all participants

		B	Std. Error	Lower	Upper	Sig.
GEE Comparison of PCS and MCS – Quality of Life (QOL) Volunteers						
PCS	T1	0^a^	0	0	0	0
	T2	−3.240	1.9506	−7.064	0.583	0.097
	T3	−1.034	3.5433	−8.858	6.790	0.776
MCS	T1	0	0	0	0	0
	T2	0.540	1.3218	−2.051	3.131	0.683
	T3	−0.616	1.3957	−3.374	2.141	0.659
GEE Comparison of PCS and MCS – Quality of Life (QOL) Older persons						
PCS	T1	0	0	0	0	0
	T2	0.300	1.5001	−2.640	3.241	0.841
	T3	−3.506	2.7585	−8.985	1.972	0.207
MCS	T1	0	0	0	0	0
	T2	1.114	1.4101	−1.653	3.881	0.430
	T3	−3.363	2.0067	−7.601	0.875	0.112
GEE Comparison of PCS and MCS – Quality of Life (QOL) Family Caregivers						
PCS	T1	0	0	0	0	0
	T2	−4.498	1.8945	−8.211	−0.785	0.018
	T3	−1.691	5.0544	−12.496	9.113	0.743
MCS	T1	0	0	0	0	0
	T2	1.000	3.0838	−5.044	7.044	0.746
	T3	4.314	4.2500	−4.020	12.649	0.310

*PCS* Physical component summary, *MCS* Mental component summary

^a^ Set to 0 because parameter is redundant

## Discussion

The purpose of this study was to evaluate the individual outcomes of a volunteer-led navigation intervention designed to provide enhanced support in the home for older persons and family living with advanced chronic illness. Qualitative evaluation indicated that older persons were satisfied with the intervention and felt it was important to their care. Specifically, older persons described the benefits they gained through enhanced social connection and support, help with negotiating the social aspect of healthcare, and identification of cost-effective resources to meet their needs. Family cited similar benefits with the addition of physical and emotional respite. Older persons desired more practical help with transportation and errands. These findings provide a more fulsome description of findings from the Nav-CARE pilot work where older persons and family cited benefits of gaining assistance with decision-making, having a social safety net, and achieving a higher level of engagement in life [51]. This discussion will contextualize our findings in relation to volunteer contributions to overall older person well-being and volunteer preparation and role satisfaction. We will further address the question of why it is so difficult for volunteer-led interventions to show statistically significant improvements in outcomes, when participants are so clearly able to articulate volunteer contributions to their care.

### Volunteer contributions to client well-being

Although there is limited evidence about the impact of the volunteer role on palliative clients and family, the evidence that does exist suggests that volunteers can increase family and client satisfaction with care and overall well-being [22, 58]. Qualitative findings from the ELSA study, a volunteer befriending service at end of life, reported that volunteers reduced negative feelings in older persons (e.g., self-pity and anxiety) and facilitated a growth in their confidence [41]. Unique findings from the Nav-CARE intervention were that volunteers helped clients to access cost effective resources and to navigate the social relationships of healthcare. Further, the volunteer navigators in this study enhanced older adult engagement. Older persons reported statistically significant improvement in their knowledge of the services available to them and confidence in their ability to make decisions about their health and healthcare. Although participants reported less confidence in making decision about their life changes and in communicating their needs and wishes to their doctor at Time 3, these items still reflected moderately high mean scores. As this was a population living with advanced chronic illness, and a number of participants died while on the study, it is possible that transitions and needs related to their illness were becoming more complex near the end of the intervention.

### Volunteer preparation and role satisfaction

Volunteers in this study felt well-prepared for their role. Several competencies revealed lower scores overall. These competencies were healthcare specific (e.g., advocating for clients with healthcare professionals or for services) and related to care of family and to community capacity building. It is possible that these were competencies that reflected the boundaries between the volunteer and professional role. So even though older persons in this study believed the volunteer navigators assisted them with navigating healthcare social relationships; this navigation may not have extended to direct advocacy with healthcare professionals. Further, the low self-perceived competence in advocating for services may have reflected the availability of services. Many of these sites were rural, and so these volunteers were likely finding themselves trying to locate and access resources that were simply not available. Although in our initial competency development we envisioned that volunteers would help to support community-capacity building, this was beyond the scope of the volunteer work in this study. In future work we will reconsider these specific competencies to determine whether volunteer training needs to be augmented or whether the competencies themselves need to be modified to further clarify the volunteer role.

Volunteers were usually satisfied with their Nav-CARE role, unless they were not paired with older persons in a timely manner. Volunteer attrition was largely related to a delay in finding older persons. The underutilization of hospice volunteers, which is largely related to a lack of knowledge about their availability and capabilities, is well-documented in the literature [59, 60]. In this study, volunteers indicated a need for further training and a desire to connect with other volunteers. Being part of a supportive community, and feeling well-prepared and mentored in their role is a critical part of volunteer satisfaction [61, 62]. In a follow-up study in progress, we are now providing both monthly coaching sessions and ongoing education in various aspects of the volunteer role (e.g., grief, boundaries, healthcare literacy).

Qualitative evaluation indicated that the volunteers valued the unique reciprocal relationship that developed between them and their clients. They derived great satisfaction from listening and learning from clients, and from assisting clients in their transitions. This relational interaction is a significant motivation for volunteering in the literature [61, 63]. Volunteers described the importance of engaging in both small talk and deeply sensitive topics, a finding that was also reported in the ELSA study [41]. Volunteers recognized the intermediary space they occupied between family and paid providers, a role that has been described elsewhere [22, 41]. However, volunteers in this study experienced challenges in relation to role fulfillment, boundary setting, and complex family dynamics. The issue of role fulfillment is one that has surfaced in all of the pilot work with Nav-CARE. The concept of navigation bears overtones of being actively engaged to make things happen. In contrast, volunteers find themselves spending what they perceive to be disproportionate amounts of time developing relationship. Further, as Nav-CARE is a new intervention it takes time for volunteers to understand and grow into the role. The complexity of volunteers dealing with family dynamics and boundary setting has been well-established in the hospice volunteer literature [62].

### Paradox of outcomes

Finally, the question arises of why there were no statistically significant improvements in volunteer, older adult, or family quality of life when this was an intervention designed to improve quality of life. Indeed, scores over time for all groups remained remarkably stable. Little is known about longitudinal patterns of quality of life in the older person upstream palliative population; although, there is some evidence to suggest that quality of life remains fairly stable at end of life, even with palliative intervention [64, 65]. A study using an intervention of a nurse navigator who collected monthly quality of life scores from 26 older persons living with advancing chronic illness, over a 4–20 month period, reflected relatively stable scores over time, although without a control group it is impossible to know what these scores would have looked like without the nurse navigator [50]. However, there is other data to suggest that those with cancer do experience diminishing quality of life compared to their counterparts with non-malignant disease [66]. In that case, a stabilization of quality of life scores might be considered a successful outcome. Without good evidence about longitudinal patterns of quality of life in specific chronic illness populations, it is difficult to know what would be a reasonable outcome when trying to improve quality of life. Further, in these longitudinal studies it is important to evaluate the magnitude of change generated by significant events so that we can better determine an appropriate effect size. A control group would have enabled us to overcome this limitation to some extent.

Older persons in this study reported comparable quality of life scores to a referent sample of individuals living with chronic disease in Australia (PCS means ranged from 36.1–46.4; MCS ranged from 47.3–50.2) [67]. For family in the present study, 47% (13/28) of scores were at or above general population norms on the PCS and 60% (17/28) scored at or above on the mental component summary. A referent sample from Canada of caregivers of persons with multiple chronic conditions provided means ranging from 48.68–48.83 for the PCS and 43.33–44.12 on the MCS [68]. Overall, reported scores were congruent with similar populations reported in the literature.

It is important to note that randomized controlled trials in both the ELSA [39, 40] and the TAPESTRY [35–38] intervention also failed to show statistically significant changes in outcomes including quality of life, social support, loneliness, self-efficacy, goal attainment, empowerment, or optimal aging. Yet, in all three of these volunteer-led interventions, qualitative results suggested that the intervention was impactful for older persons and family. The challenge is, of course, that it is difficult to influence policy based upon qualitative evidence when randomized controlled trials fail to support the effectiveness of the intervention. It is possible that the essential differences that volunteers were making in these interventions were not captured well within the measurement instruments used.

A recently published scoping review identified four primary outcomes from supplementary palliative support services: enriching relationships, greater autonomy and perceived control, knowing more, and improved mental health [69]. This review further revealed how many commonly used outcome measures fail to include all four domains. In the context of this study, the SF12v2 addressed only the improved mental health domain whereas the older adult engagement questionnaire addressed all four domains. However, the engagement questionnaire still failed to show statistically significant improvements in most areas even when older persons described very specific gains they realized in being involved with these volunteers (e.g., better decisions, emotional respite, low cost services). It is possible that these four domains are influenced by many factors beyond volunteer contributions (e.g., family, healthcare providers) and so it is difficult to demonstrate the unique contribution of volunteers independent of these other factors. Here is where the qualitative work becomes particularly important in developing a more nuanced understanding of impact. There is now a developing qualitative body of evidence that will facilitate the construction and testing of measurements specific to what might be realized through volunteer-led interventions [41, 62].

This study has several limitations that should be considered when evaluating the findings. A number of older persons died while on the study, and there were significant delays in recruiting older persons in some communities. The difficulties in recruiting older persons were related to a number of factors including public perceptions of hospice, professional gatekeeping, and a concern that recruitment strategies would identify too many older persons and thus overwhelm the capacity of the organization. These factors have been described in detail in another paper [52]. This meant that not all older persons received the full 12- or 18-month intervention. Further, this meant that some volunteers went for long periods after the training without a client, which in turn had implications for the collection of self-efficacy scores. These scores were then more useful in identifying where volunteers felt less prepared and less useful in providing a representation of developing competency. The lack of psychometric data on the engagement questionnaire limits the generalizability of the findings. Future research will conduct psychometric testing of this questionnaire. Finally, there was no control group by which to compare the measured impacts.

## Conclusion

This study contributes to a growing body of evidence on how innovative volunteer models can improve care for older persons living at home with chronic illness. Building upon a palliative approach to care, this volunteer-led navigation intervention (Nav-CARE) has the potential to improve social support, access to low-cost resources, healthcare social negotiation and engagement of older persons living at home with advancing chronic illness. Volunteers provided physical and emotional respite for family. Volunteers were well-prepared for, and satisfied in, the Nav-CARE role as long as they were matched with older persons in a timely manner. Further, research needs to be done in developing volunteer-sensitive outcomes so that volunteer contributions can be documented more robustly. The study authors are in the process of scaling out the Nav-CARE intervention to additional sites across Canada to build a more robust understanding of its potential impact.

### Ethics approval and consents to participate

This study was approved by the Behaviour Research Ethics Board of the University of British Columbia. [H16–02265 & H16–02304] the University of Alberta [Pro00070579] and Dalhousie University [2016–4039]. All participants provided written, signed consent.

## Supplementary information

**Additional file 1: Supplementary File 1.** Older person engagement questionnaire. The file provides the questionnaire used to assess the outcome of older person engagement.

**Additional file 2: Supplementary File 2.** Volunteer navigator self-efficacy questionnaire. This file provides the questionnaire used by volunteers to self-report their confidence on the 32 competencies addressed in the volunteer navigation training.

**Additional file 3: Supplementary File 3.** Volunteer navigator satisfaction questionnaire. This file provides the questionnaire used by volunteers to report their satisfaction with various aspects of the Nav-CARE intervention.

**Additional file 4: Supplementary File 4.** Older person quality of life scores: not imputed. This file provides the results of the older person quality of life scores for non-imputed data.

## Data Availability

The datasets generated and/or analysed during the current study are not publicly available for privacy reasons but are available from the corresponding author on reasonable request.

## Abbreviations

ELSA: End-of-Life Action Study
Nav-CARE: Navigating: Connecting, Accessing, Resourcing, Engaging
QOL: Quality of Life
GEE: Generalized Estimating Eq.
MI: Multiple Imputation
MCMC: Markov Chain Monte Carlo
PCS: Physical Component Summary
MCS: Mental Component Summary
P: Older Person
V: Volunteer
F: Family

## Footnotes

1 The study was funded by two agencies with different funding lengths. One study focused on older adults living with a variety of chronic illness (18 months). The other study focused on a cancer specific population (12 months). The intervention and evaluation were congruent across studies. Data were pooled to increase study power.

## References

[1] Albrecht H, Comartin J, Valeriote F, Block K, Scarpaleggia F (2011). Not to be Forgotten: Care of Vulnerable Canadians.

[2] The Canadian Medical Association (2013). Health and health Care for an Aging Population.

[3] World Health Organization (2018). Ageing and Health.

[4] Stajduhar K (2011). Chronic illness, palliative care, and the problematic nature of dying. Can J Nurs Res.

[5] Haggerty JL (2012). Ordering the chaos for patients with multimorbidity. Br Med J.

[6] Mason B, Nanton V, Epiphaniou E, Murray SA, Donaldson A, Shipman C, Daveson BA, Harding R, Higginson IJ, Munday D, Barclay S, Dale J, Kendall M, Worth A, Boyd K (2016). ‘My body is falling apart.’ Understanding the experiences of patients with advanced multimorbidity to improve care: Serial interviews with patients and carers. BMJ Support.

[7] Mason B, Epiphaniou E, Nanton V, Donaldson A, Shipman C, Daveson BA, Harding R, Higginson I, Munday D, Barclay S, Boyd K, Dale J, Kendall M, Worth A, Murray SA (2013). Coordination of care for individuals with advanced progressive conditions: a multi-site ethnographic and serial interview study. Br J Gen Pract.

[8] Wajnberg A, Ornstein K, Zhang M, Smith KL, Soriano T (2013). Symptom burden in chronically ill homebound individuals. J Am Geriatr Soc.

[9] Eckerblad J, Theander D, Ekdahl A, Unosson M, Wirehn AB, Milberg A, Krevers B, Jaarsma T (2015). Symptom burden in community-dwelling older people with multi-morbidity: a cross-sectional study. BMC Geriatr.

[10] Walke L, Gallo W, Tinetti M, Fried T (2004). The burden of symptoms among community-dwelling olderpersons with advanced chronic disease. Arch..

[11] National seniors council: Report on the social isolation of seniors. 2014. https://www.canada.ca/content/dam/nsc-cna/documents/pdf/policy-and-program-development/publications-reports/2014/Report_on_the_Social_Isolation_of_Seniors.pdf. Accessed 19 Feb 2020.

[12] Murray S, Boyd K, Kendall M, Worth A, Benton T, Clausen H (2002). Dying of lung cancer or cardiac failure: prospective qualitative interview study of patients and their carers in the community. BMJ..

[13] Gallagher LP, Truglio-Londrigan M (2004). Community support: older adults’ perceptions. Clin Nurs Res.

[14] Booth S, Fallon M, Hollis G (2016). Rhetoric and reality - matching palliative care services to meet the needs of patients of all ages, with any diagnosis. Palliat Med.

[15] Moss AH, Lunney JR, Culp S, Auber M, Kurian S, Rogers J, Dower J, Abraham J (2010). Prognostic significance of the “surprise” question in cancer patients. J Palliat Med.

[16] Sawatzky R, Porterfield P, Lee J, Dixon D, Lounsbury K, Pesut B, Roberts D, Tayler C, Voth J, Stajduhar K. Conceptual foundations of a palliative approach: A knowledge synthesis. BMC Palliat Care. 2016. 10.1186/s12904-016-0076-9. 10.1186/s12904-016-0076-9 PMC4715271 26772180

[17] Sawatzky R, Porterfield P, Roberts D, Lee J, Liang L, Reimer-Kirkham S, Pesut B, Schalkwyk T, Stajduhar K, Tayler C, Baumbusch J, Thorne S (2016). Embedding a palliative approach in nursing care delivery: an integrated knowledge synthesis.

[18] Shadd JD, Burge F, Stajduhar KI, Cohen SR, Kelley ML, Pesut B (2013). Defining and measuring a palliative approach in primary care. Can Fam Physician.

[19] Stajduhar K, Tayler C (2014). Taking an “upstream” approach in the care of dying cancer patients: the case for a palliative approach. Can Oncol Nurs J.

[20] Burge F, Lawson B, Mitchell G (2012). When and how to take a palliative approach to care for people with multimorbidity. BMJ..

[21] Gridley K, Brooks J, Glendinning C (2014). Good practice in social care for disabled adults and older people with severe and complex needs: evidence from a scoping review. Health Soc Care Comm.

[22] Candy B, France R, Lowa J, Sampson L (2015). Does involving volunteers in the provision of palliative care make a difference to patient and family well-being? A systematic review of quantitative and qualitative evidence. Int J Nurs Stud.

[23] Canadian Hospice Palliative Care Association, Government of Canada, Quality end-of-life Care Coalition of Canada: Integrating a Palliative Approach into the Management of Chronic, Life-threatening Diseases: Who, How and When? 2013; http://hpcintegration.ca/media/38753/TWF-palliative-approach-report-English-final2.pdf. Accessed 13 Feb 2020.

[24] Goossensen A, Somsen J, Scott R, Pelttari L (2016). Defining volunteering in hospice and palliative care in Europe: an EPAC white paper. Eur J Palliat Care.

[25] Claxton-Oldfield S, Claxton-Oldfield J (2008). Some common problems faced by hospice palliative care volunteers. Am J Hosp Palliat Me.

[26] Fillion L, Cook S, Veillette AM, Aubin MI, de Serres M, Rainville FO, Fitch M, Doll R (2012). Professional navigation framework: elaboration and validation in a Canadian context. Oncol Nurs Forum.

[27] Fillion L, Cook S, Veillette AM, de Serres M (2012). Aubin ml, Rainville FO, Fitch M, Doll R. professional navigation: a comparative study of two Canadian models. Can Oncol Nurs J..

[28] Rodday AM, Parsons SK, Snyder F, Simon MA, Lianos AA, Warren-Mears V, Dudley D, Lee JH, Patierno SR, Markossian TW, Sanders M, Whitley EM, Freund KM (2015). Impact of patient navigation in eliminating economic disparities in cancer care. Cancer..

[29] Freund KM, Barttaglia T, Calhoun EA, Darnell J, Dudley D, Fiscella K (2014). Writing group of the patient navigation research program: impact of patient navigation on timely cancer care: the patient navigation research program. J Natl Cancer I.

[30] Lorhan S, Cleghorn L, Fitch M, Pang K, McAndrew A, Applin-Poole J, Ledwell E, Mitchell R, Wright M (2013). Moving the agenda forward for cancer patient navigation: understanding volunteer and peer navigation approaches. J Cancer Educ.

[31] Balaban RB, Galbraith A, Burns M, Vialle-Valentin C, Larochelle M, Ross-Degnan D (2015). A patient navigator intervention to reduce hospital readmissions among high-risk safety-net patients: a randomized controlled trial. J Gen Intern Med.

[32] Robinson CA, Pesut B, Bottorff JL (2010). Issues in rural palliative care: views from the countryside. Jrn Rural Health.

[33] Duggleby WD, Penz K, Leipert BD, Wilson DM, Goodridge D, Williams A (2011). 'I am part of the community but...' The changing context of rural living for persons with advanced cancer and their families. Rural Rem Health.

[34] Robinson CA, Pesut B, Bottorff JL (2012). Supporting rural family palliative caregivers. Jrn Fam Nurs.

[35] Dolovich L, Oliver D, Lamarche L, Thabane L, Valaitis R, Agarwal G, Carr T, Foster G, Griffith L, Javadi D, Kastner M, Mangin D, Papaioannou A, Ploeg J, Raina P, Richardson J, Risdon C, Santaguida P, Straus S, Price D (2019). Combining volunteers and primary care teamwork to support health goals and needs of older adults: a pragmatic randomized controlled trial. Can Med Assoc J.

[36] Kastner M, Sayal R, Oliver D, Straus SE, Dolovich L (2017). Sustainability and scalability of a volunteer-based primary care intervention: a mixed-methods analysis. BMC Health Serv Res.

[37] Dolovic L, Oliver D, Lamarche L, Agarwal G, Carr T, Chan D, Cleghorn L, Griffith L, Javadi D, Kastner M, Longaphy J, Mangin D, Papaioannou A, Ploeg J, Raina P, Richardson J, Risdon C, Santaguida PL, Straus S, Thabane L, Valaitis R, Price D. A protocol for a pragmatic randomized controlled trial using the Health Teams Advancing Patient Experience: Strengthening quality platform approach to promote person-focused primary healthcare for older adults. Implement Sci. 2016. 10.1186/s13012-016-0407-5. 10.1186/s13012-016-0407-5 PMC4820854 27044360

[38] Ploeg J, Valaitis RK, Cleghorn L, Yous M-L, Gaber J, Agarwal G, Kastner M, Mangin D, Oliver D, Parascandalo F, Risdon C, Dolovich L. Perceptions of older adults in Ontario, Canada on the implementation and impact of a primary care programme: a descriptive qualitative study. BMJ Open. 2019. 10.1136/bmjopen-2018-026257. 10.1136/bmjopen-2018-026257 PMC6575818 31201187

[39] Walshe C, Algorta GP, Dodd S, Hill M, Ockenden N, Payne S, Preston N (2016). Protocol for the end-of-life social action study (ELSA): a randomised wait-list controlled trial and embedded qualitative case study evaluation assessing the causal impact of social action befriending services on end of life experience. BMC Palliat Care.

[40] Walshe C, Dodd S, Hill M, Ockenden N, Payne S, Preston N, Perez AG (2016). How effective are volunteers at supporting people in their last year of life? A pragmatic randomised wait-list trial in palliative care. BMC Med.

[41] Dodd S, Payne S, Preston N, Walshe C, Hill M, Ockenden N, Algorta GP (2018). 'Being with' or 'doing for'? How the role of an end-of-life volunteer befriender can impact patient wellbeing: interviews from a multiple qualitative case study (ELSA). Supp Care Canc.

[42] Pesut B, Robinson CA, Bottorff JL. Among Neighbours: an ethnographic account of responsibilities in rural palliative care. Palliat Support Care. 2013. 10.1017/S1478951512001046A. 10.1017/S1478951512001046 23510757

[43] Pesut B, Bottorff JL, Robinson CA (2011). Be known, be available, be mutual: a qualitative ethical analysis of social values in rural palliative care. BMC Med Ethics.

[44] Duggleby WD, Penz K, Leipert BD, Wilson DM, Goodridge D, Williams A (2011). 'I am part of the community but...' The changing context of rural living for persons with advanced cancer and their families. Rural Remote Health.

[45] Duggleby WD, Penz KL, Goodridge DM, Wilson DM, Leipert BD, Berry PH, Keall SR, Justice CJ (2010). The transition experience of rural older persons with advanced cancer and their families: a grounded theory study. BMC Palliat Care.

[46] Duggleby W, Cooper L, Leipert B, Wilson D, Williams A, Marshall D, Goodridge D (2012). Development of a “changes toolkit” for rural older palliative patients and their family caregivers. J Rural Community Dev.

[47] Duggleby W, Anderson J, Baxter S, Berry P, Cooper D, Faainsinger R, Fassbender K, Fraser K, Gayman K, Ghosh S, Goodridge D, Hallstrom L, Kaasalainen S, Kary S, Keating N, Kenmore K, MacLeod R, Mann A, Nekolaichuk C, Pesut B, Peterson Fraser M, Robinson C, Santos Salas A, Swanson S, Swindle J, Watanabe S, Watson L, Whitfield K, Williams A, Woytkiw T (2014). Which Way from Here? Navigation Competencies for the Care of Older Rural Adults at the End of Life.

[48] Duggleby W, Robinson CA, Kaasalainen S, Pesut B, Nekolaichuk C, MacLeod R, Keating NC, Santos Salas A, Hallstrom LK, Fraser KD, Williams A, Struthers Montford K, Swindle K (2016). Developing navigation competencies to care for older rural adults with advanced illness. Can J Aging.

[49] Duggleby W, Pesut B, Cottrell L, Friesen L, Sullivan K, Warner G (2018). Development, implementation, and evaluation of a curriculum to prepare volunteer navigators to support older persons living with serious illness. Am J Hosp Palliat Care..

[50] Pesut B, Hooper B, Jacobsen M, Nielsen B, Dalhuisen M, O'Connor B (2017). Nurse-led navigation to provide early palliative care in rural areas: a pilot study. BMC Palliat Care..

[51] Pesut B, Duggleby W, Warner G, Antifeau E, Hooper B, Greig M, Sullivan K (2017). Volunteer navigation partnerships: a compassionate community approach to early palliative care. BMC Palliat Care.

[52] Pesut B, Duggleby W, Warner G, Kervin E, Bruce P, Antifeau E, Hooper B (2020). Implementing volunteer navigation for older persons with advanced chronic illness (Nav-CARE): a knowledge to action study. BMC Palliative Care.

[53] Cresswell JW, Plano Clark VL (2011). Designing and conducting mixed methods research.

[54] Pascuet E, Beauchemin L, Vaillancourt R, Cowin L, Ni A, Rattray M (2012). Volunteer satisfaction and program evaluation at a pediatric hospice. J Palliative Med.

[55] Ware J, Kosinski M, SD K. (1996). A 12-item short form health survey: construction of scales and preliminary tests of reliability and validity. Med Care.

[56] Cheak-Zamora N, Wyrwich K, TD M. Reliability and validity of the SF-12v2 in the medical expenditure panel survey. Qual Life Res. 2009;18(6):727-35. 10.1007/s11136-009-9483-1 19424821

[57] Sandelowski M (2010). What's in a name? Qualitative description revisited. Res Nurs Health.

[58] Burbeck R, Candy B, Low J, Rees R (2014). Understanding the role of the volunteer in specialist palliative care: a systematic review and thematic synthesis of qualitative studies. BMC Palliat Care.

[59] Whittall D, Lee S, O'Connor M (2016). Factors affecting rural volunteering in palliative care - an integrated review. Aust J Rural Health.

[60] Claxton-Oldfield S (2015). Got volunteers? The selection, training, roles, and impact of hospice palliative care volunteers in Canada’s community-based volunteer programs. Home Health Care Manag Pract.

[61] Söderhamn U, Flateland S, Fensli M, Skaar R (2017). To be a trained and supported volunteer in palliative care - a phenomenological study. BMC Palliat Care..

[62] Pesut B, Hooper B, Lehbauer S, Dalhuisen M. Promoting volunteer capacity in hospice palliative care: a narrative review. Am J Hosp Palliat Care. 2012. 10.1177/1049909112470485. 10.1177/1049909112470485 23277631

[63] Muckaden MA, Pandya SS (2016). Motivation of volunteers to work in palliative care setting: a qualitative study. Indian J Palliat Care.

[64] Hermann CP, Looney SW (2011). Determinants of quality of life in patients near the end of life: a longitudinal perspective. Oncol Nurs Forum.

[65] Connell T, Griffiths R, Fernandez RS, Griffiths R, Tran D, Agar M, Harlum J (2011). Quality-of-life trajectory of clients and carers referred to a community palliative care service. Int J Palliat Nurs.

[66] Walshe C, Preston N, Payne S, Dodd S, Perez AG (2018). Quality of life trends in people with and without cancer referred to volunteer-provided palliative care services (ELSA): a longitudinal study. J Pain Symptom Manag.

[67] Jayasinghe UW, Proudfoot J, Barton CA, Amoroso C, Holton C, Davies GP, Beilby J, Harris MF. Quality of life of Australian chronically-ill adults: patient and practice characteristics matter. Health Qual Life Out. 2009. 10.1186/1477-7525-7-50. 10.1186/1477-7525-7-50 PMC2700088 19493336

[68] Duggleby W, Williams A, Ghosh S, Moquin H, Ploeg J, Markle-Reid M, Peacock S (2016). Factors influencing changes in health related quality of life of caregivers of persons with multiple chronic conditions. Health Qual Life Out..

[69] Dodd SR, Payne SA, Preston NJ, Walshe CE. Understanding the outcomes of supplementary support services in palliative care for older people. A scoping review and mapping exercise. J Pain Symptom Manage. 2020;60(2):449-59e21. 10.1016/j.jpainsymman.2020.03.010 32201310

