# Supplementary material for: A mixed-method evaluation of a volunteer navigation intervention for older persons living with chronic illness (Nav-CARE): findings from a knowledge translation study
Source: BMC Palliat Care. 2020 Oct 15;19:159. doi: 10.1186/s12904-020-00666-2 (PMC7565322; doi:10.1186/s12904-020-00666-2)
Supplement: Supplementary file 1 — Additional file 1: Supplementary File 1. Older person engagement questionnaire. The file provides the questionnaire used to assess the outcome of older person engagement. [file 12904_2020_666_MOESM1_ESM.docx]

Supplementary File 1: Older Person Engagement Questionnaire

1. I feel I know the services available in my community to help me.

| **All of the time** 1 □ | **Most of the time** 2 □ | **Some of the time** 3 □ | **A little of the time** 4 □ | **None of the time** 5 □ |
| --- | --- | --- | --- | --- |

1. I feel like I have people to turn to when I need help.

| **All of the time**   1 □ | **Most of the time** 2 □ | **Some of the time** 3 □ | **A little of the time** 4 □ | **None of the time** 5 □ |
| --- | --- | --- | --- | --- |

1. I feel lonely.

| **All of the time**  1 □ | **Most of the time** 2 □ | **Some of the time** 3 □ | **A little of the time** 4 □ | **None of the time** 5 □ |
| --- | --- | --- | --- | --- |

1. I feel I can be involved in the things that are important to me.

| **All of the time**  1 □ | **Most of the time** 2 □ | **Some of the time** 3 □ | **A little of the time** 4 □ | **None of the time** 5 □ |
| --- | --- | --- | --- | --- |

1. I feel I have someone I can talk to about the things that are troubling me.

| **All of the time**  1 □ | **Most of the time** 2 □ | **Some of the time** 3 □ | **A little of the time** 4 □ | **None of the time** 5 □ |
| --- | --- | --- | --- | --- |

1. I feel confident in making decisions about my life changes.

| **All of the time**  1 □ | **Most of the time** 2 □ | **Some of the time** 3 □ | **A little of the time** 4 □ | **None of the time** 5 □ |
| --- | --- | --- | --- | --- |

1. I know where to get information about my illness.

| **All of the time**  1 □ | **Most of the time** 2 □ | **Some of the time** 3 □ | **A little of the time** 4 □ | **None of the time** 5 □ |
| --- | --- | --- | --- | --- |

1. I feel confident in taking care of my illness.

| **All of the time**  1 □ | **Most of the time** 2 □ | **Some of the time** 3 □ | **A little of the time** 4 □ | **None of the time** 5 □ |
| --- | --- | --- | --- | --- |

1. I am confident contacting someone when I have a health problem.

| **All of the time**  1 □ | **Most of the time** 2 □ | **Some of the time** 3 □ | **A little of the time** 4 □ | **None of the time** 5 □ |
| --- | --- | --- | --- | --- |

1. I understand the information given to me by my doctor and other healthcare providers.

| **All of the time**  1 □ | **Most of the time** 2 □ | **Some of the time** 3 □ | **A little of the time** 4 □ | **None of the time** 5 □ |
| --- | --- | --- | --- | --- |

1. I feel confident making decisions about my health and healthcare.

| **All of the time**  1 □ | **Most of the time** 2 □ | **Some of the time** 3 □ | **A little of the time** 4 □ | **None of the time** 5 □ |
| --- | --- | --- | --- | --- |

1. I feel confident communicating my needs and wishes to my doctor and other healthcare providers.

| **All of the time**  1 □ | **Most of the time** 2 □ | **Some of the time** 3 □ | **A little of the time** 4 □ | **None of the time** 5 □ |
| --- | --- | --- | --- | --- |
