# Supplementary material for: A mixed-method evaluation of a volunteer navigation intervention for older persons living with chronic illness (Nav-CARE): findings from a knowledge translation study
Source: BMC Palliat Care. 2020 Oct 15;19:159. doi: 10.1186/s12904-020-00666-2 (PMC7565322; doi:10.1186/s12904-020-00666-2)
Supplement: Supplementary file 2 — Additional file 2: Supplementary File 2. Volunteer navigator self-efficacy questionnaire. This file provides the questionnaire used by volunteers to self-report their confidence on the 32 competencies addressed in the volunteer navigation training. [file 12904_2020_666_MOESM2_ESM.docx]

Supplementary File 2: Volunteer Navigator Self-Efficacy Questionnaire.

*For each of the following statements please circle the number that best describes how confident you feel:*

| ***I feel confident in my ability to:*** | ***Not at all***  ***confident*** | | | | ***Highly***  ***confident*** | | |
| --- | --- | --- | --- | --- | --- | --- | --- |
| 1. Know when to access my volunteer coordinator | 0 | 1 | 2 | 3 | | 4 | 5 |
| 1. Assess client/family quality of life concerns | 0 | 1 | 2 | 3 | | 4 | 5 |
| 1. Identify client/family values and beliefs regarding end of life issues | 0 | 1 | 2 | 3 | | 4 | 5 |
| 1. Prioritize with client/family concerns across quality of life areas | 0 | 1 | 2 | 3 | | 4 | 5 |
| 1. Determine client/family preferred engagement in decision making and self-navigation | 0 | 1 | 2 | 3 | | 4 | 5 |
| 1. Identify client/family knowledge and perception of available options and community resources | 0 | 1 | 2 | 3 | | 4 | 5 |
| 1. Determine if there is a need for community resources and services | 0 | 1 | 2 | 3 | | 4 | 5 |
| 1. Identify client/family needs for resources | 0 | 1 | 2 | 3 | | 4 | 5 |
| 1. Assist family caregivers in care provision decisions | 0 | 1 | 2 | 3 | | 4 | 5 |
| 1. Identify barriers to needed resources | 0 | 1 | 2 | 3 | | 4 | 5 |
| 1. Advocate to meet client/family needs with healthcare professionals | 0 | 1 | 2 | 3 | | 4 | 5 |
| 1. Assist client/family to overcome service access barriers | 0 | 1 | 2 | 3 | | 4 | 5 |
| 1. Advise client/family on negotiating for care and services | 0 | 1 | 2 | 3 | | 4 | 5 |
| 1. Facilitate strategies for self-navigation | 0 | 1 | 2 | 3 | | 4 | 5 |
| 1. Perform an environmental scan for community services, care providers, and events | 0 | 1 | 2 | 3 | | 4 | 5 |
| 1. Identify community assets for client/family | 0 | 1 | 2 | 3 | | 4 | 5 |
| 1. Identify best-fit for client/family with community resources | 0 | 1 | 2 | 3 | | 4 | 5 |
| 1. Create linkages to local leaders, professionals and resources | 0 | 1 | 2 | 3 | | 4 | 5 |
| 1. Assist client/family to build and connect (or reconnect) with networks/connections | 0 | 1 | 2 | 3 | | 4 | 5 |
| 1. Identify client/family concern and/or needs | 0 | 1 | 2 | 3 | | 4 | 5 |
| 1. Assess client/family need for support | 0 | 1 | 2 | 3 | | 4 | 5 |
| 1. Develop plans reflective of client/family needs and concerns | 0 | 1 | 2 | 3 | | 4 | 5 |
| 1. Provide family with caregiving support and resources | 0 | 1 | 2 | 3 | | 4 | 5 |
| 1. Coordinate access to needed services | 0 | 1 | 2 | 3 | | 4 | 5 |
| 1. Assess client/family service usage | 0 | 1 | 2 | 3 | | 4 | 5 |
| 1. Facilitate beginning discussion with client/family about advanced care planning (ACP) and goals of care | 0 | 1 | 2 | 3 | | 4 | 5 |
| 1. Assist client/family with access to services for loss, grief and bereavement support | 0 | 1 | 2 | 3 | | 4 | 5 |
| 1. Determine effective and appropriate ways to meet client needs | 0 | 1 | 2 | 3 | | 4 | 5 |
| 1. Identify level of desired client/family involvement | 0 | 1 | 2 | 3 | | 4 | 5 |
| 1. Build capacity with client/family towards their desired level of independence and engagement | 0 | 1 | 2 | 3 | | 4 | 5 |
| 1. Promote engagement in decision-making and end of life issues by client/family | 0 | 1 | 2 | 3 | | 4 | 5 |
| 1. Evaluate continuously the level of client/family engagement in decision-making and end of life issues | 0 | 1 | 2 | 3 | | 4 | 5 |
