# Supplementary material for: A mixed-method evaluation of a volunteer navigation intervention for older persons living with chronic illness (Nav-CARE): findings from a knowledge translation study
Source: BMC Palliat Care. 2020 Oct 15;19:159. doi: 10.1186/s12904-020-00666-2 (PMC7565322; doi:10.1186/s12904-020-00666-2)
Supplement: Supplementary file 3 — Additional file 3: Supplementary File 3. Volunteer navigator satisfaction questionnaire. This file provides the questionnaire used by volunteers to report their satisfaction with various aspects of the Nav-CARE intervention. [file 12904_2020_666_MOESM3_ESM.docx]

Supplementary File 3: Volunteer Navigator Satisfaction Questionnaire

1. **SECTION B - Part I ORIENTATION**

|  | Strongly Disagree | Disagree | No Opinion | Agree | Strongly Agree | Does not apply |
| --- | --- | --- | --- | --- | --- | --- |
| My orientation to the NCARE volunteer program was sufficient (e.g., understanding how the program worked). |  |  |  |  |  |  |
| My orientation prepared me to fill out the visit paperwork. |  |  |  |  |  |  |
| My orientation to my assigned client was sufficient. |  |  |  |  |  |  |
| My orientation to community resources was sufficient. |  |  |  |  |  |  |

1. **SECTION B - Part II TRAINING**

|  | Strongly Disagree | Disagree | No Opinion | Agree | Strongly Agree | Does not apply |
| --- | --- | --- | --- | --- | --- | --- |
| My training as an NCARE volunteer was sufficient. |  |  |  |  |  |  |
| Following my training, I felt prepared to be an NCARE volunteer. |  |  |  |  |  |  |
| My training was offered in a timely fashion. |  |  |  |  |  |  |
| Following my training, I was comfortable to begin work. |  |  |  |  |  |  |
| My training prepared me well for the emotional challenges of my position. |  |  |  |  |  |  |
| My training prepared me well for appropriate communication with clients. |  |  |  |  |  |  |
| My training prepared me well for appropriate communication with family members. |  |  |  |  |  |  |
| I do not need any further training to continue work as an NCARE volunteer. |  |  |  |  |  |  |

1. **SECTION B - Part III FEEDBACK / PERFORMANCE**

|  | Strongly Disagree | Disagree | No Opinion | Agree | Strongly Agree | Does not apply |
| --- | --- | --- | --- | --- | --- | --- |
| If I have questions about my NCARE volunteer work I feel comfortable to ask. |  |  |  |  |  |  |
| If I have questions, someone is available to answer them. |  |  |  |  |  |  |
| I receive adequate feedback from the NCARE volunteer coordinator. |  |  |  |  |  |  |
| The feedback I receive from the NCARE volunteer coordinator is constructive. |  |  |  |  |  |  |
| The feedback I receive from the NCARE volunteer coordinator is useful. |  |  |  |  |  |  |
| I receive adequate feedback from other NCARE volunteers. |  |  |  |  |  |  |
| The feedback I receive from NCARE volunteers is constructive. |  |  |  |  |  |  |
| The feedback I receive from NCARE volunteers is useful. |  |  |  |  |  |  |
| Overall, I receive adequate feedback about my performance as an NCARE volunteer. |  |  |  |  |  |  |

1. **SECTION B - Part IV COMMUNICATION**

|  | Strongly Disagree | Disagree | No Opinion | Agree | Strongly Agree | Does not apply |
| --- | --- | --- | --- | --- | --- | --- |
| I am well informed about the medical needs of the NCARE clients. |  |  |  |  |  |  |
| I am well informed about the social needs of the NCARE clients. |  |  |  |  |  |  |
| I am well informed about broader hospice issues in my community. |  |  |  |  |  |  |
| I am well informed about hospice events. |  |  |  |  |  |  |
| Good communication exists between the NCARE volunteer coordinator, hospice and myself. |  |  |  |  |  |  |
| Good communication exists between the NCARE volunteer coordinator, other health care professionals and myself. |  |  |  |  |  |  |
| Good communication exists between other NCARE volunteers and myself. |  |  |  |  |  |  |

1. **SECTION B - Part V SOCIAL CONTACTS**

|  | Strongly Disagree | Disagree | No Opinion | Agree | Strongly Agree | Does not apply |
| --- | --- | --- | --- | --- | --- | --- |
| I know other NCARE volunteers well. |  |  |  |  |  |  |
| I would like the opportunity to have more social contacts with NCARE volunteers. |  |  |  |  |  |  |
| I know other hospice volunteers well. |  |  |  |  |  |  |
| I would like the opportunity to have more social contacts with my local hospice. |  |  |  |  |  |  |

1. **SECTION B - Part VI VALUE AND RESPECT**

|  | Strongly Disagree | Disagree | No Opinion | Agree | Strongly Agree | Does not apply |
| --- | --- | --- | --- | --- | --- | --- |
| I am a valued member of the NCARE program. |  |  |  |  |  |  |
| I feel my work, as an NCARE volunteer, is important. |  |  |  |  |  |  |
| I feel my work, as an NCARE volunteer, is needed. |  |  |  |  |  |  |
| I help improve the quality of life for the NCARE clients. |  |  |  |  |  |  |
| My skills as an NCARE volunteer are used to their full potential. |  |  |  |  |  |  |
| My volunteer position is what I expected it to be. |  |  |  |  |  |  |
| I am happy with my volunteer position. |  |  |  |  |  |  |
| Communication with the NCARE volunteer coordinator is respectful. |  |  |  |  |  |  |
| Communication with healthcare staff is respectful. |  |  |  |  |  |  |
| Communication with other hospice staff is respectful. |  |  |  |  |  |  |
| Communication with other volunteer navigators is respectful. |  |  |  |  |  |  |

Thank you for your feedback.

*This survey was adapted with permission from a survey used as part of a volunteer satisfaction and program evaluation at Roger’s House: Pascuet, E., Beauchemin, L., Vaillancourt, R., Cowin, L., Ni, A., & Rattray, M. (2012). Volunteer satisfaction and program evaluation at a pediatric hospice. Journal of Palliative Medicine, 15(5), 567-572*
