# Supplementary material for: A mixed-method evaluation of a volunteer navigation intervention for older persons living with chronic illness (Nav-CARE): findings from a knowledge translation study
Source: BMC Palliat Care. 2020 Oct 15;19:159. doi: 10.1186/s12904-020-00666-2 (PMC7565322; doi:10.1186/s12904-020-00666-2)
Supplement: Supplementary file 4 — Additional file 4: Supplementary File 4. Older person quality of life scores: not imputed. This file provides the results of the older person quality of life scores for non-imputed data. [file 12904_2020_666_MOESM4_ESM.docx]

Supplementary File 4: Older Person Quality of Life Scores: Not Imputed

|  |  | Beta | Std Error | Lower | Upper | Sig. |
| --- | --- | --- | --- | --- | --- | --- |
| PCS | T1 | 0 | 0 | 0 | 0 | 0 |
|  | T2 | 0.479 | 1.5386 | -2.537 | 3.494 | 0.756 |
|  | T3 | -3.173 | 2.5454 | -8.162 | 1.816 | 0.213 |
| MCS |  |  |  |  |  |  |
|  | T1 | 0 | 0 | 0 | 0 | 0 |
|  | T2 | 1.109 | 1.3678 | -1.572 | 3.79 | 0.417 |
|  | T3 | -3.145 | 1.9092 | -6.887 | 0.597 | 0.099 |

PCS: Physical Component Summary

MCS: Mental Component Summary

T1: n=34. T2: n=33. T3: n=20

Older Person Engagement Item Scores: Not imputed

|  |  | B | std error | lower | upper | sig |
| --- | --- | --- | --- | --- | --- | --- |
| Q1 | T1 | 0 | 0 | 0 | 0 | 0 |
|  | T2 | -0.514 | 0.2022 | -0.911 | -0.118 | 0.011 |
|  | T3 | -0.777 | 0.2036 | -1.176 | -0.378 | <0.0001 |
| Q2 | T1 | 0 | 0 | 0 | 0 | 0 |
|  | T2 | -0.217 | 0.1821 | -0.574 | 0.139 | 0.232 |
|  | T3 | -0.27 | 0.1723 | -0.608 | 2.455 | 0.117 |
| Q3 | T1 | 0 | 0 | 0 | 0 | 0 |
|  | T2 | 0.272 | 0.1621 | -0.146 | 0.59 | 0.093 |
|  | T3 | -0.133 | 0.2412 | -0.606 | 0.34 | 0.581 |
| Q4 | T1 | 0 | 0 | 0 | 0 | 0 |
|  | T2 | 0.157 | 0.2205 | -0.275 | 0.589 | 0.476 |
|  | T3 | 0.513 | 0.274 | -0.024 | 1.05 | 0.061 |
| Q5 | T1 | 0 | 0 | 0 | 0 | 0 |
|  | T2 | -0.047 | 0.1813 | -0.403 | 0.308 | 0.794 |
|  | T3 | 0.092 | 0.2242 | -0.348 | 0.531 | 0.683 |
| Q6 | T1 | 0 | 0 | 0 | 0 | 0 |
|  | T2 | -0.042 | 0.1697 | -0.375 | 0.29 | 0.803 |
|  | T3 | 0.152 | 0.22 | -0.279 | 0.583 | 0.49 |
| Q7 | T1 | 0 | 0 | 0 | 0 | 0 |
|  | T2 | -0.162 | 0.1053 | -0.368 | 0.045 | 0.124 |
|  | T3 | 0.113 | 0.2419 | -0.361 | 0.587 | 0.641 |
| Q8 | T1 | 0 | 0 | 0 | 0 | 0 |
|  | T2 | -0.288 | 0.1563 | -0.594 | 0.019 | 0.066 |
|  | T3 | 0.028 | 0.222 | -0.408 | 0.463 | 0.901 |
| Q9 | T1 | 0 | 0 | 0 | 0 | 0 |
|  | T2 | -0.061 | 0.1197 | -0.296 | 0.174 | 0.61 |
|  | T3 | 0.256 | 0.2159 | -0.167 | 0.679 | 0.235 |
| Q10 | T1 | 0 | 0 | 0 | 0 | 0 |
|  | T2 | -0.357 | 0.1817 | -0.713 | -0.001 | 0.049 |
|  | T3 | 0.035 | 0.2361 | -0.428 | 0.498 | 0.882 |
| Q11 | T1 | 0 | 0 | 0 | 0 | 0 |
|  | T2 | -0.225 | 0.1293 | -0.478 | 0.029 | 0.082 |
|  | T3 | 0.294 | 0.2068 | -0.111 | 0.699 | 0.155 |
| Q12 | T1 | 0 | 0 | 0 | 0 | 0 |
|  | T2 | -0.175 | 0.1494 | -0.468 | 0.118 | 0.242 |
|  | T3 | -0.046 | 0.2256 | -0.488 | 0.396 | 0.838 |
